# Changes in Infectious Disease–Specific Health Literacy in the Post–COVID-19 Pandemic Period: Two-Round Cross-Sectional Survey Study

**DOI:** 10.2196/52666

**Published:** 2024-08-30

**Authors:** Yusui Zhao, Yue Xu, Dingming Yao, Qingqing Wu, Heni Chen, Xiujing Hu, Yu Huang, Xuehai Zhang

**Affiliations:** 1 Zhejiang Provincial Center for Disease Control and Prevention, Hangzhou, China

**Keywords:** survey, infectious disease–specific health literacy, COVID-19, health education, factors, postpandemic

## Abstract

**Background:**

Infectious disease–specific health literacy (IDSHL) is a crucial factor in the development of infectious diseases. It plays a significant role not only in mitigating the resurgence of infectious diseases but also in effectively averting the emergence of novel infections such as COVID-19. During the 3 years of the COVID-19 pandemic, China primarily adopted nonpharmaceutical interventions, advocating for people to avoid crowded places and wear masks to prevent the spread of COVID-19. Consequently, there has been a dearth of research concerning IDSHL and its corresponding focal points for health education.

**Objective:**

This study aimed to (1) evaluate the changes in IDSHL scores between 2019 (before the COVID-19 pandemic) and 2022 (the postepidemic period of COVID-19) and (2) explore the risk factors affecting IDSHL using a multivariate logistic regression analysis.

**Methods:**

This study used 2-round cross-sectional surveys, conducted in 2019 and 2022, respectively, in 30 counties in Zhejiang Province, China. Multiple-stage stratified random sampling was used to select households, and a Kish grid was used to identify participants. An identical standardized questionnaire consisting of 12 closed-ended questions was used to measure IDSHL scores before and after the COVID-19 pandemic (2019 and 2022). Standard descriptive statistics, chi-square tests, *t* tests, and multivariate logistic regression analyses were used to analyze the data.

**Results:**

The 2-round cross-sectional surveys conducted in 2019 and 2022 yielded, out of 19,366 and 19,221 total questionnaires, 19,257 (99.44% response rate) and 18,857 (98.11% response rate) valid questionnaires, respectively. The correct response rate for the respiratory infectious diseases question “When coughing or sneezing, which of the following is correct?” increased from 29.10% in 2019 to 37.92% in 2022 (*χ*²_1_=332.625; *P*<.001). The correct response rate for the nonrespiratory infectious diseases question “In which of the following ways can hepatitis B be transmitted to others?” decreased from 64.28% to 59.67% (*χ*²_1_=86.059; *P*<.001). In terms of IDSHL scores, a comparison between 2022 and 2019 revealed notable statistical differences in the overall scores (*t*_1_=10.829; *P*<.001) and across the 3 dimensions of knowledge (*t*_1_=8.840; *P*<.001), behavior (*t*_1_=16.170; *P*<.001), and skills (*t*_1_=9.115; *P*<.001). With regard to the questions, all but 4 exhibited statistical differences (*P*<.001). Multivariate logistic regression analyses indicated that the 2022 year group had a higher likelihood of possessing acquired IDSHL than the 2019 group (odds ratio 1.323, 95% CI 1.264‐1.385; *P*<.001).

**Conclusions:**

When conducting health education, it is imperative to enhance efforts in nonrespiratory infectious disease health education, as well as respiratory infectious diseases such as COVID-19. Health education interventions should prioritize ethnic minority populations with a poor self-health status and low education.

## Introduction

In the final months of 2019, a contagious disease caused by SARS-CoV-2 was discovered in Wuhan, China, and rapidly spread throughout the country and then globally [1]. From the end of 2019 to the end of 2022, China went through 4 stages [2,3]. The first stage was an emergency response and blockage stage (from the initial outbreak to March 2020). It took approximately 3 months to achieve decisive results in the defense of Wuhan, successfully blocking the domestic transmission of the disease. The second stage consisted of exploring routine prevention and control measures (April 2020 to July 2021). This stage focused on expanding prevention and control measures through nucleic acid testing, controlling the disease within 2 or 3 incubation periods. The third stage was the “dynamic zero” stage of comprehensive and precise prevention and control along the entire chain (August 2021 to February 2022). The goal of this stage was to minimize the occurrence of the disease, efficiently handle scattered cases and clusters, and control the disease within 1 incubation period (14 days) by attempting to achieve the greatest prevention and control effect at the smallest social cost. The fourth stage was the comprehensive prevention and control stage of “scientific precision, dynamic zero” (March 2022 to December 2022). During this stage, in addition to emphasizing rapid and precise prevention and control, comprehensive prevention and control measures were emphasized, including the management of infectious sources, rapid blocking of transmission routes, and protection of susceptible populations. These measures were effectively combined and stacked to prevent the spread of the disease. With the occurrence of viral mutations, changes in the epidemic, access to vaccinations, and the accumulation of prevention and control experience and capabilities, China’s prevention and control of the epidemic entered a new stage—on January 8, 2023, that is, 3 years after China’s COVID-19 epidemic prevention and control campaign began, COVID-19 entered the fifth stage, that is, management [4]. China had thus entered the post–COVID-19 era.

Research indicated that the application of vaccines has notably diminished the prevalence of COVID-19 infections within the population to a measurable degree [5]. Apart from vaccinations, nonpharmacological interventions (NPIs) were the most important measures to prevent the spread of the COVID-19 pandemic [6-10], such as maintaining social distancing, wearing masks, washing hands frequently, airing rooms frequently, and taking care when sneezing. The pervasive implementation of vaccines and NPIs was intrinsically linked to public cognition. Only when individuals possessed infectious disease–specific health literacy (IDSHL) and had a comprehensive understanding of COVID-19 did they proactively seek vaccination or adopt NPIs to prevent infection. Consequently, this heightened awareness empowered individuals to actively pursue vaccination or adopt NPIs as strategies to prevent infection of COVID-19 [11]. IDSHL emphasized 3 key components—cognition, decision-making abilities, and self-efficacy, all of which are essential for the prevention and treatment of infectious diseases [12]. Since the outbreak of the pandemic, the National Health Commission of China has updated and published its “Novel Coronavirus Infection Prevention and Control Protocol” 10 times [11], emphasizing that “everyone is responsible for their own health” and for maintaining good hygiene habits such as frequent hand washing, the wearing of masks, strengthening of personal protection, and ongoing promotion of education and awareness. IDSHL and NPIs have indeed played a positive role in effectively preventing COVID-19 infections among Chinese residents. The COVID-19 pandemic has been effectively controlled in China, and the incidence of respiratory and gastrointestinal diseases has significantly decreased [6,13]. This has led to feelings of both satisfaction and concern. In the post–COVID-19 era, when the country no longer requires the public to wear masks and maintain social distancing, it remains to be seen whether the incidence of respiratory and gastrointestinal diseases will continue its downward trend.

Zhejiang, a province in southeastern China, had a population of 64 million at the end of 2020. As a large province, it has seen the second-largest influx of migrant workers in China. These individuals relocated from their homes and now reside in cramped living conditions with inadequate sanitation. They lack many basic rights, such as open access to employment opportunities, free education, social welfare programs, and medical benefits. These conditions significantly increase the risk of outbreaks of infectious diseases. IDSHL is an important determinant of such outbreaks [14]. The lower an individual’s level of IDSHL, the more likely they are to contract a disease and experience poorer outcomes [15,16]. This suggests that measuring the changes in IDSHL before and after the pandemic may make it possible to predict, with some accuracy, any future changes in the incidence of respiratory and gastrointestinal diseases among residents. However, few studies have explored how IDSHL has changed since the pandemic.

Therefore, the purpose of this study was to (1) evaluate the changes IDSHL scores between 2019 (before the COVID-19 pandemic) and 2022 (the postepidemic period of COVID-19) and (2) explore the risk factors affecting IDSHL among residents using a multivariate logistic regression analysis.

## Methods

### Study Design

This study used 2 cross-sectional surveys, conducted in 2019 and 2022, respectively, in 30 counties in Zhejiang Province, China.

### Ethical Considerations

This study adhered to the principles of the Declaration of Helsinki. Informed consent was obtained from all participants or their legal guardians, and all survey responses were collected anonymously. In appreciation of their participation, all participants were presented with a modest gift valued at 50 RMB (about US $7) upon the conclusion of the survey. This study was approved by the ethics committee of the Zhejiang Provincial Center for Disease Control and Prevention (2022-027-01).

### Sampling and Recruitment Procedure

The sample size for each county was calculated using the formula:


$$N=\frac{\mu_{\alpha }^{2}\times p\left(1-p\right)}{\delta^{2}}\times deff$$

where α (.05) represents the significance level, µ_α_ (1.96) is the α-quantile of the standard normal distribution, *p* (26.24%, based on the health literacy level of Zhejiang Province residents in 2018) is the health literacy level, δ (0.03936) is the maximum permissible error, and *deff* (1) is the design effect of complex sampling. Following the exclusion of invalid questionnaires and rejections (25%), the final sample size for each county was 640. The total sample size of the 30 counties was 19,200. The same sampling method and recruitment procedures were used for both cross-sectional surveys. Multiple-stage stratified random sampling was used to select the participants. Based on the hierarchical administrative system and 2010 Chinese Census data, sampling recruitment procedures were conducted in five stages: (1) 30 counties were selected from the 90 counties in Zhejiang Province, (2) 4 townships were selected within each county, (3) 2 segments (residential blocks) were selected within each township, (4) 100 households were selected within each segment based on a complete list of the addresses of all households, and (5) 1 participant was selected from each household using a Kish grid. Once the ultimate sample outcomes were ascertained, the subsequent recruitment and investigation of participants were exclusively executed by community workers or community health physicians (investigators). First, they contacted the sampled household head by phone, informed them of the family member to be surveyed, and then scheduled a face-to-face visit. If the appointment failed, another one was scheduled. If 3 consecutive attempts to schedule a successful visit failed or recruitment was unsuccessful, we proceeded with the survey by moving on to the next household on the sampling list, and recruitment was restarted. The sampling frame was derived from the 2010 Chinese Census data and field mapping. The eligibility criteria were (1) aged 15‐69 years, (2) able to read or communicate, and (3) accessible to the researchers.

### Measures

A battery of instruments was used to measure the participants’ IDSHL and collect sociodemographic data (Multimedia Appendix 1). IDSHL was assessed using a subscale of the health literacy surveillance survey questionnaire developed by the National Health Commission of China [17]. The subscale addresses the knowledge, behavior, and skills related to infectious disease prevention and control. It consists of one true or false item, 8 single-choice items, and 3 multiple-choice items. Each correct response to a multiple-choice question receives 2 points, with 1 point for each correct response to a single-choice or judgment question. The total score for the questionnaire is 15 points. If the respondents scored 12 points or higher, we assumed that they had acquired IDSHL. This subscale is reliable and widely used in China [18]. Each of the 12 items had a content validity index >0.8, and the overall Cronbach α coefficient was 0.67.

We also collected participants’ sociodemographic data including their sex, age, ethnicity, education, marital status, occupation, and self-health status. A 5-point Likert-type scale was used to assess self-health status (1=excellent, 2=very good, 3=good, 4=fair, and 5=poor).

### Data Analysis

Statistical analyses were performed using SPSS (version 18.0; IBM Corp). The mean (SD) and frequency were calculated to describe the quantitative and qualitative variables, respectively. Chi-square tests were used to determine the statistical differences in the demographic characteristics and in each item relating to infectious diseases between the 2019 and 2022 groups. *2-tailed t* tests (Welch *F* test) were used to determine the differences in demographic characteristics and the 3 dimensions of the IDSHL score between the 2 groups. Logistic regression analysis was used to explore the risk factors affecting IDSHL among the participants. A score of *P*<.05 was considered statistically significant.

The independent variables that were included in all models are sex (male=0 and female=1); age, in years (18‐29=1, 30‐39=2, 40‐49=3, and 50‐69=4); ethnicity (Han=1 and minority=2); education (primary school or lower=1, middle school=2, high school=3, technical school or college=4, and undergraduate or higher=5); marital status (unmarried, divorced, or widowed=0 and married=1); occupation (farmers=1, workers=2, agency or institutional personnel=3, students=4, and other=5); self-health status (excellent=1, very good=2, good=3, fair=4, and poor=5); and year (2019=1 and 2022=2).

## Results

### Sociodemographic Characteristics of the Groups

A total of 19,366 individuals were surveyed in 2019, with 19,257 valid questionnaires (response rate: 19,257/19,366, 99.44%), and 19,221 individuals were surveyed in 2022, with 18,857 valid questionnaires (response rate: 18,857/19,221, 98.11%). Table 1 presents a comparison of the demographic and health-related characteristics between the 2 groups surveyed in 2019 (n=19,257) and 2022 (n=18,857). There were no statistically significant differences in ethnicity or marital status between the 2 groups, with the vast majority of individuals identifying as Han Chinese (98.93% in 2019 and 98.82% in 2022; *P*=.32) and married (83.31% in 2019 and 83.23% in 2022; *P*=.84). However, there were statistically significant differences between the 2019 and 2022 groups with regard to sex (*P*=.02), age (*P*<.001), education (*P*<.001), occupation (*P*<.001), and self-health status (*P*<.001; Table 1). Given the disparities in certain sociodemographic characteristics between the 2 groups, particularly factors such as age and education, which substantially influence IDSHL, there was a potential for the outcomes of the 2 groups to be noncomparable. To ensure the comparability of the results, we standardized both groups using age and education based on the entire surveyed population (N=38,114).

**Table 1.** Sociodemographic characteristics of participants in the 2019 and 2022 groups.

Content and group		2019 group (n=19,257), n (%)	2022 group (n=18,857), n (%)	Chi-square (*df*)	*P* value
**Sex**				5.3 (1)	.02
	Male	9175 (47.65)	8762 (46.47)		
	Female	10,082 (52.35)	10,095 (53.53)		
**Age (years)**				144.6 (3)	<.001
	18‐29	1370 (7.23)	1529 (8.26)		
	30‐39	2313 (12.21)	2626 (14.19)		
	40‐49	2749 (14.51)	3218 (17.39)		
	50‐69	12,513 (66.05)	11,130 (60.15)		
**Ethnicity**				1.0 (1)	.32
	Han	19,050 (98.93)	18,634 (98.82)		
	Minority	207 (1.07)	223 (1.18)		
**Education**				167.9 (4)	<.001
	Primary school or lower	7020 (36.45)	5887 (31.22)		
	Middle school	6196 (32.18)	6254 (33.17)		
	High school	3130 (16.25)	3134 (16.62)		
	Technical school or college	2848 (14.79)	3480 (18.45)		
	Undergraduate or higher	63 (0.33)	102 (0.54)		
**Marital status**				0.1 (1)	.84
	Unmarried, divorced, or widowed	3214 (16.69)	3162 (16.77)		
	Married	16,043 (83.31)	15,695 (83.23)		
**Occupation**				186.7 (4)	<.001
	Farmers	8738 (45.38)	7600 (40.3)		
	Workers	2581 (13.4)	2681 (14.22)		
	Agency or institutional personnel^a^	1927 (10.01)	1945 (10.31)		
	Students	671 (3.48)	442 (2.34)		
	Other^b^	5340 (27.73)	6189 (32.82)		
**Self-health status** ^c^				40.8 (4)	<.001
	Poor	244 (1.27)	175 (0.93)		
	Fair	815 (4.23)	644 (3.42)		
	Good	6242 (32.41)	5906 (31.32)		
	Very good	5823 (30.24)	5773 (30.61)		
	Excellent	6133 (31.85)	6359 (33.72)		
Total		19,257 (100)	18,857 (100)	—^d^	—

a “Agency or institutional personnel” refers to people working in state organizations, state-owned enterprises, institutions, and other public roles.

b The “Other” category includes unemployed people and those with occupations other than those already listed.

c “Self-health status” refers to respondents’ perceived health status in the preceding 12 months.

d Not applicable.

### Comparison of Correct Response Rates for Specific Questions

The data indicated that, in general, the percentage of correct answers increased from 2019 to 2022 for most questions. The single-choice questions saw a statistically significant increase in the percentage of individuals who answered correctly in 2022 compared with 2019 for 3 out of the 8 questions (*P*<.001). The multiple-choice questions saw a significant increase in the percentage of individuals who answered correctly in 2022 compared with 2019 for all 3 questions (*P*<.001; Table 2).

**Table 2.** Comparison of correct rates for each specific question of IDSHL^a^ questionnaire between 2019 and 2022 groups.

Types and question		Answered correctly (2019), n (%)	Answered correctly (2022), n (%)	Chi-square (*df*)	*P* value
**True or false question**					
	The best way to prevent flu is to take antibiotics (anti-inflammatories).	10,880 (57.06)	10,793 (56.66)	0.6 (1)	.43
**Single-choice questions**					
	In which of the following ways can hepatitis B be transmitted to others?	12,257 (64.28)	11,366 (59.67)	86.1 (1)	<.001
	For the treatment of tuberculosis patients, which of the following statements is correct?	12,126 (63.59)	12,088 (63.46)	0.1 (1)	.78
	In which of the following situations should vaccination of children be suspended?	15,514 (81.36)	15,977 (83.87)	41.9 (1)	<.001
	If you have a fever, which of the following is correct?	15,031 (78.83)	15,811 (83.01)	107.5 (1)	<.001
	If a virulent infectious disease occurs in a certain place, which of the following practices is correct?	15,800 (82.86)	16,502 (86.63)	104.6 (1)	<.001
	Open windows frequently for ventilation during flu season. Regarding window ventilation, which of the following statements is correct?	14,117 (74.04)	14,061 (73.82)	0.2 (1)	.63
	What is the correct way to read body temperature with a glass thermometer?	10,115 (53.05)	10,576 (55.52)	23.5 (1)	<.001
	If you are bitten by a dog but not seriously, what is the right thing to do?	18,509 (97.07)	18,474 (96.99)	0.2 (1)	.64
**Multiple-choice questions**					
	What should parents do when their children have symptoms such as fever and a rash?	13,099 (68.70)	13,673 (71.78)	43.4 (1)	<.001
	When sick and dead livestock are found, which of the following practices is correct?	14,818 (77.71)	15,185 (79.72)	22.9 (1)	<.001
	When coughing or sneezing, which of the following is correct?	5549 (29.10)	7223 (37.92)	332.6 (1)	<.001

a IDSHL: infectious disease–specific health literacy.

### Comparison of IDSHL Scores by Sociodemographic Characteristics

Both males and females showed statistically significant improvements (*P*<.001) in scores. All age groups also showed statistically significant improvements (*P*<.001) with the youngest age group (18-29 years) having the highest scores in both years. The Han group showed a significant improvement in IDSHL scores (*P*<.001), whereas there was no significant change for ethnic minority groups (*P*=.95). Education level was also a statistically significant factor, with higher levels of education being associated with greater improvements in IDSHL scores (*P*<.001) for all groups except primary school or lower (*P*=.08).

Marital status and occupation were also associated with IDSHL score improvements. Unmarried, divorced, or widowed participants and those in certain occupations (agency or institutional personnel, students, and others) showed statistically significant improvements in their IDSHL scores (*P*<.001). By contrast, there was no statistically significant change in IDSHL scores for those who reported poor, fair, and good self-health status (*P*=.995, *P*=.094, and *P*=.03, respectively), whereas participants with very good and excellent self-health status showed statistically significant improvements (*P*<.001 for all; Table 3).

**Table 3.** Comparison of IDSHL^a^ scores of participants by sociodemographic characteristics between the 2019 and 2022 groups.

Content and group		2019 survey group (n=19,257), mean (SD)	2022 survey group (n=18,857), mean (SD)	*t* test (*df*)	*P* value
**Sex**					
	Male	10.04 (3.13)	10.37 (3.16)	7.145 (1)	<.001
	Female	10.03 (3.19)	10.40 (3.24)	8.136 (1)	<.001
**Age (years)**					
	18‐29	11.61 (2.55)	12.23 (2.35)	6.808 (1)	<.001
	30‐39	11.51 (2.69)	12.16 (2.47)	8.796 (1)	<.001
	40‐49	10.85 (2.93)	11.26 (2.86)	5.565 (1)	<.001
	50‐69	9.28 (3.15)	9.52 (3.20)	5.813 (1)	<.001
**Ethnicity**					
	Han	10.04 (3.16)	10.39 (3.20)	10.845 (1)	<.001
	Minority	8.87 (2.59)	8.96 (4.75)	0.066 (1)	.95
**Education**					
	Primary school or lower	8.60 (3.09)	8.69 (3.19)	1.743 (1)	.08
	Middle school	9.88 (3.08)	10.33 (2.97)	8.180 (1)	<.001
	High school	11.04 (2.66)	11.57 (2.61)	7.967 (1)	<.001
	Technical school or college	12.19 (2.24)	12.70 (2.03)	9.516 (1)	<.001
	Undergraduate or higher	12.15 (2.29)	12.98 (1.65)	2.701 (1)	.01
**Marital status**					
	Unmarried, divorced, or widowed	11.06 (2.98)	11.58 (2.88)	5.279 (1)	<.001
	Married	9.98 (3.15)	10.33 (3.19)	9.958 (1)	<.001
**Occupation**					
	Farmers	9.19 (3.16)	9.33 (3.28)	2.739 (1)	.01
	Workers	9.81 (3.11)	10.18 (3.07)	4.263 (1)	<.001
	Agency or institutional personnel	11.49 (2.72)	12.35 (2.39)	10.477 (1)	<.001
	Students	11.50 (2.57)	12.11 (2.35)	4.000 (1)	<.001
	Other	10.69 (3.01)	11.14 (2.88)	8.252 (1)	<.001
**Self-health status**					
	Poor	8.01 (3.27)	8.01 (3.15)	0.006 (1)	.995
	Fair	9.00 (3.28)	9.29 (3.33)	1.678 (1)	.10
	Good	9.87 (3.07)	9.93 (3.26)	1.100 (1)	.30
	Very good	10.52 (3.11)	10.96 (3.01)	7.717 (1)	<.001
	Excellent	9.94 (3.20)	10.48 (3.18)	9.585 (1)	<.001

a IDSHL: infectious disease–specific health literacy.

### Comparison of IDSHL Scores for 3 Dimensions

Table 4 presents data related to the health knowledge, behavior, and skills dimensions for the 2-year groups. The results show a statistically significant improvement in the mean scores for all the 3 dimensions between 2019 and 2022. The knowledge dimension showed a statistically significant increase (*P*<.001), with mean scores of 4.22 (SD 1.60) in 2019 and 4.43 (SD 1.60) in 2022. The behavioral dimension also showed a statistically significant increase (*P*<.001), with mean scores of 4.46 (SD 1.66) in 2019 and 4.73 (SD 1.69) in 2022. The skills dimension showed a statistically significant increase (*P*<.001) with mean scores of 1.36 (SD 0.68) in 2019 and 1.42 (SD 0.66) in 2022 (Table 4).

**Table 4.** Comparison of knowledge, behavior, and skill scores of participants between the 2019 and 2022 groups.

Subscale	2019 group, mean (SD)	2022 group, mean (SD)	*t* test (*df*)	*P* value
Knowledge	4.22 (1.60)	4.43 (1.60)	8.840（1）	<.001
Behavior	4.46 (1.66)	4.73 (1.69)	16.170（1）	<.001
Skills	1.36 (0.68)	1.42 (0.66)	9.115（1）	<.001
Overall	10.03 (3.16)	10.38 (3.20)	10.829（1）	<.001

### Multivariate Logistic Regression Analyses

To estimate the effect sizes of these possible risk factors, we conducted a multivariate logistic regression analysis. Table 5 shows the B, SE, Wald test, *P* value, and odds ratio (OR; 95% CI) values for the potential risk factors. In the multivariate logistic regression model of acquired IDSHL, sex, age, ethnicity, education, marital status, occupation, self-health status, and year group were identified as risk factors. Education is strongly associated with IDSHL. Middle school (OR 2.155, 95% CI 2.028‐2.290), high school (OR 3.590, 95% CI 3.323‐3.879), technical school or college (OR 7.399, 95% CI 6.727‐8.139), and undergraduate or higher education (OR 12.919, 95% CI 8.400‐19.870) were associated with higher IDSHL scores than a primary school education or lower. Self-health status was strongly associated with IDSHL, with a better self-health status being associated with higher IDSHL. Compared with the 2019 group, the 2022 group was more likely to have acquired IDSHL (OR 1.323, 95% CI 1.264‐1.385).

**Table 5.** Multivariate logistic regression analyses to characterize risk factors associated with IDSHL^a^ of participants.

Variables		β (SE)		Wald	*P* value	OR^b^ (95% CI)	
**Sex**							
	Male (Reference)	0.065 (0.024)		7.500	.01	1.067 (1.019‐1.118)	
**Age**							
	18‐29 (Reference)	N/A^c^		N/A	N/A	N/A	
	30‐39	−0.047 (0.064)		0.553	.46	0.954 (0.842‐1.081)	
	40‐49	−0.052 (0.065)		0.632	.43	0.949 (0.835‐1.079)	
	50‐69	−0.528 (0.063)		69.310	<.001	0.590 (0.521‐0.668)	
**Ethnicity**							
	Minority (Reference)	0.249 (0.115)		4.660	.03	1.283 (1.023‐1.608)	
**Education**							
	Primary school or lower (Reference)	N/A		N/A	N/A	N/A	
	Middle school	0.768 (0.031)		612.469	<.001	2.155 (2.028‐2.290)	
	High school	1.278 (0.039)		1051.036	<.001	3.590 (3.323‐3.879)	
	Technical school or college	2.001 (0.049)		1697.265	<.001	7.399 (6.727‐8.139)	
	Undergraduate or higher	2.559 (0.220)		135.729	<.001	12.919 (8.400‐19.870)	
**Marital status**							
	Unmarried, divorced, or widowed (Reference)	0.248 (0.038)		43.362	<.001	1.281 (1.190‐1.379)	
**Occupation**							
	Farmers (Reference)	N/A		N/A	N/A	N/A	
	Workers	0.002 (0.037)		0.002	.97	1.002 (0.932‐1.076)	
	Agency or institutional personnel	0.23 (0.048)		23.416	<.001	1.259 (1.147‐1.382)	
	Students	0.228 (0.087)		6.905	.01	1.256 (1.060‐1.490)	
	Other	0.148 (0.031)		23.230	<.001	1.160 (1.092‐1.232)	
**Self-health status**							
	Poor (Reference)	N/A		N/A	N/A	N/A	
	Excellent	0.628 (0.147)		18.299	<.001	1.874 (1.406‐2.500)	
	Very good	0.82 (0.147)		31.145	<.001	2.271 (1.702‐3.029)	
	Good	0.632 (0.147)		18.565	<.001	1.882 (1.411‐2.509)	
	Fair	0.508 (0.159)		10.244	.001	1.661 (1.217‐2.267)	
**Year**							
	2019 (Reference)	0.280 (0.023)		143.296	<.001	1.323 (1.264‐1.385)	
Constant		−2.658 (0.200)		176.160	<.001	0.041 (0.025-0.073)	

a IDSHL: infectious disease–specific health literacy.

b OR: odds ratio.

c N/A: not applicable.

## Discussion

### Principal Findings

In the 21st century, China has faced 2 major challenges in the realm of infectious diseases—the resurgence of previously prevalent diseases [19] and the emergence of new infectious diseases [20]. China was among the first countries to detect and respond to the COVID-19 outbreak, implementing a robust infectious disease surveillance system. Leveraging this system, China was able to swiftly and effectively mobilize resources to control the spread of COVID-19, resulting in the successful containment of the epidemic within a relatively brief period [21]. The full application of the concept of the human destiny community provides useful insights into changes in global public health governance [22]. In general, efforts have been made to enhance the availability and accessibility of global public health products while concurrently fostering international collaboration, and despite a growing population, the incidence, morbidity, and mortality rates of infectious diseases have decreased since 2000 [23]. The decline in the incidence, morbidity, and mortality rates of infectious diseases in China can be attributed to the country’s ongoing efforts to enhance its infectious disease surveillance system and to its continuous health education initiatives aimed at promoting healthy lifestyle behaviors and improving the public’s IDSHL. As a social determinant of health [24], IDSHL is known to affect health behaviors, health outcomes, communication with providers, adherence to treatment regimens, and health care costs. Therefore, improving IDSHL is crucial for effective prevention efforts.

In our study, we described the changes in IDSHL scores over time among residents of Zhejiang Province, China, based on representative 2-time-series survey data before and after the outbreak of the COVID-19 pandemic. Our comparative analysis of the sociodemographic characteristics between the 2 groups revealed that the surveyed population in 2022 exhibited higher educational attainment and relatively younger age than the 2019 group. To address this issue and ensure comparability of results, we performed a standardization of the 2 population groups, adjusting for age and education. In addition, the difference could be ascribed to progress in social and economic development, with the implementation of NPIs contributing substantially to the substantial enhancement of residents’ IDSHL [25]. IDSHL plays a crucial role in mediating the relationship between background data and preventive behaviors [26]. Therefore, it is of utmost importance to consider IDSHL when designing public interventions. A crucial factor contributing to the success of China’s response to the COVID-19 pandemic was the implementation of prompt and decisive measures by the Chinese government. In the early stages of the outbreak, China effectively used robust containment strategies, resulting in a significant reduction in the number of confirmed COVID-19 cases [27]. When localized outbreaks emerged, stringent measures, such as rapid nucleic acid testing and rigorous control over transportation, were swiftly enforced, effectively curtailing the spread of the virus.

NPIs reduced the incidence of non–COVID-19 infectious diseases effectively [28], particularly respiratory infections during the COVID-19 pandemic [29]. Analyzing specific questions from our survey, the most significant increase in correct response rates between 2022 and 2019 was observed for the question “When coughing or sneezing, which of the following is correct?” The correct response rate increased for this question from 29.10% to 37.92%. Similarly, notable improvements were seen in the questions “If a virulent infectious disease occurs in a certain place, which of the following practices is correct?” and “If you have a fever, which of the following is correct?” The correct response rates increased from 82.86% and 78.83%, respectively, in 2019, to 86.63% and 83.01%, respectively, in 2022. These findings provide further empirical evidence that aligns with the findings of previous studies. One noteworthy observation is that the correct response rate for the question “In which of the following ways can hepatitis B be transmitted to others?” decreased from 64.28% in 2019 to 59.67% in 2022. This result seems to contradict China’s infectious disease surveillance data [30]. This apparent contradiction may be explained by the fact that China has placed a greater emphasis on COVID-19 prevention and control in recent years, leading to a relaxation in the promotion of preventive measures for nonrespiratory infectious diseases, such as hepatitis B. Consequently, residents’ knowledge regarding the prevention and control of such diseases has declined. We speculate that the decrease in hepatitis B incidence was a result of the stringent isolation and control measures implemented by the government during the pandemic. These measures inadvertently caused some asymptomatic carriers of hepatitis B, who might have been detected while seeking medical attention for other illnesses, to remain undetected, thereby resulting in a potential underestimation of the incidence of hepatitis B. This provides an important reminder that once the COVID-19 pandemic was over, the incidence of nonrespiratory infectious diseases was not only unlikely to continue decreasing but could in fact see a noticeable increase.

This study compared the IDSHL scores of participants with different demographic characteristics in 2 surveys. The findings revealed a greater disparity in IDSHL between Han and ethnic minority groups over the period. Moreover, the ethnic minority groups’ IDSHL scores did not exhibit significant improvement during the 2 survey periods. These findings, which are consistent with research conducted by Tuohetamu et al [31], suggest that the observed disparity in IDSHL among ethnic minority groups in Zhejiang Province may be attributable more to their low education and income than to language barriers alone [32]. Education has emerged as one of the most critical factors affecting IDSHL [33,34]. In this study, participants with a primary school education or lower were found to have the lowest IDSHL scores, and no substantial enhancement in their scores was observed across the 2 survey iterations. Plausible explanations for this phenomenon stem from their constrained cognitive capabilities, limited aptitude for learning, and diminished capacity to absorb new information, leading to poor IDSHL acquisition. This study did not see a significant increase in the IDSHL scores of participants who reported poor, fair, and good self-health from 2019 to 2022, suggesting that health education practitioners should try targeted health intervention measures to improve the IDSHL of residents with relatively poor self-health status [35].

In the multivariate logistic regression analysis, after adjusting for factors such as sex, age, ethnicity, education, marital status, and self-health status, the year group was found to be one of the influencing factors affecting IDSHL. Considering the IDSHL scores and the rates of correct responses to the questions in both surveys, it is evident that there was a significant improvement in participants’ IDSHL in 2022 compared with 2019, following the 3-year COVID-19 pandemic. This improvement can be primarily attributed to the notable enhancement in residents’ knowledge, behaviors, and skills pertaining to the prevention and management of respiratory infectious diseases [6]. The decline in knowledge about nonrespiratory infectious diseases, however, suggests that, while it is important to reinforce health education among residents regarding respiratory infectious diseases, it is equally important to enhance health education pertaining to nonrespiratory infectious diseases.

### Limitations

This study has some limitations. First, the representativeness of our study population compared with the general Chinese population may have been affected by our sampling strategy. Second, it is important to acknowledge that our study used cross-sectional surveys conducted at 2 different time points, which may have introduced a potential selection bias into our sample. Third, given the cross-sectional nature of this study, it was not possible to determine causation. Therefore, we cannot conclude that IDSHL increased because of the increase in COVID-19. Finally, our research population consisted of permanent residents aged 15‐69 years, and some groups were not included; such groups should be included in subsequent studies.

### Conclusions

We observed a significant improvement in participants’ IDSHL in Zhejiang Province after 3 years of the COVID-19 pandemic, especially in terms of knowledge and behaviors related to respiratory infectious disease prevention and control. However, we also noticed a decline in the correct response rates for nonrespiratory infectious diseases, such as hepatitis B. Therefore, we believe it is necessary to strengthen health education efforts for nonrespiratory infectious diseases alongside the ongoing education on COVID-19 and other respiratory infectious diseases. We recommend that a provincial infectious disease surveillance system be fully used to monitor infectious diseases in the province, which will enable further research on the relationship between IDSHL and the occurrence of infectious diseases among residents. In addition, to address health disparities and promote equity, health education interventions should prioritize ethnic minority populations in the province with a relatively poor self-health status and low education.

## Supplementary material

10.2196/52666 Multimedia Appendix 1 Questionnaire on Infectious-Disease-Specific Health Literacy.

## Abbreviations

IDSHL: infectious disease–specific health literacy
NPI: nonpharmacological intervention
OR: odds ratio
