# Supplementary material for: Changes in Infectious Disease–Specific Health Literacy in the Post–COVID-19 Pandemic Period: Two-Round Cross-Sectional Survey Study
Source: JMIR Public Health Surveill. 2024 Aug 30;10:e52666. doi: 10.2196/52666 (PMC11378864; doi:10.2196/52666)
Supplement: Multimedia Appendix 1 [file publichealth-v10-e52666-s001.docx]

Questionnaire on Infectious-Disease-Specific Health Literacy

coding：□□□□□□□

**Instructions for filling out the questionnaire**

Please fill in the box after each question with the serial number of the option you selected, such as:

2. Gender： ①male  ②female 
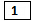


**Introduction and informed consent**

Hello, we are the staff of Zhejiang Provincial Center for Disease Control and Prevention. In order to understand your infectious disease-specific health literacy, we need to ask you a few questions, please provide some personal information, contact information, answer some questions about the prevention and treatment of infectious diseases. This information will be only to facilitate the future to find you and give you scientific health guidance. The content involved in the investigation will be kept confidential for you and will not be disclosed to any third party. So please answer truthfully. Participation in this survey will be completely voluntary, and you may refuse to answer any questions you are not willing to answer, and you can terminate the survey at any time if you wish to do so in the survey. By participating in this survey, you can acquire knowledge and information on the prevention of infectious diseases, and help relevant departments formulate strategies and measures for the prevention and control of infectious diseases. So as to effectively prevent infectious diseases and better protect everyone. The survey will take you about 10 minutes. I will give you a small souvenir after the survey. If you agree to participate in the survey, please sign and cooperate with us to complete the survey.

Thank you for your support!

**Respondent's signature after informed consent:**

**Sociodemographic characteristics**

1.**Name**：

1. **Gender**：  ①male     ②female  **□**
2. **Age**：_________

4.**Contact information**

Mobile：_________

5.**Ethnicity**：  ①Han ②Minority **□**

6.**Education**：  **□**

①Primary school or lower    ②Middle school     ③High school

④Technical school or college     ⑤Undergraduate or higher

7. **Marital status**：   **□**

①Unmarried/divorced/widow ②Married

8. **Occupation**：  **□**

①Farmers ②Workers

③Agency/ Institutional personnel  ④Students

⑤Other

1. Total household income/year：_________¥

Family population：

10.What do you think of your physical condition： **□**

①Excellent ②Very good   ③Good

④Fair ⑤Poor

**IDSHL Questionnaire**

1. The best way to prevent flu is to take antibiotics (anti-inflammatories). **□**

①Agree  ②Disagree ③Don't know

2.Which of the following ways can hepatitis B be transmitted to others? **□**

①Work, eat, and swim with sick or infected people

②It can be transmitted through sex, blood transfusion and mother-to-child transmission

③Talk to, shake hands with, and hug a sick or infected person

④Don't know

3.For the treatment of tuberculosis patients, which of the following statements is correct? **□**

①No preferential policies

②The state provides anti-TB drugs free of charge

③Free hospitalization

④Don't know

4. In which of the following situations should vaccination of children be suspended? **□**

①When you cry

②When you have a cold or fever

③Within half an hour after a meal

④Don't know

5. If you have a fever, which of the following is correct? **□**

①Timely seek medical attention

②Based on past experience, take over-the-counter fever medication

③Observe and see

④Don't know

6. If a virulent infectious disease occurs in a certain place, which of the following practices is correct? **□**

①This illness has nothing to do with me, so I don't need to pay attention to it

②If I were a local, I would pay attention to the epidemic situation

③Whether or not I am a local, I need to pay attention to the changing epidemic situation

④Don't know

7. Open windows frequently for ventilation during flu season. Regarding window ventilation, which of the following statements is correct? **□**

①In winter, it is necessary to open windows less or not at all to avoid catching a cold

②Opening windows for ventilation can dilute bacteria and viruses in indoor air

③Opening windows can allow sunlight into the room, which can kill various bacteria and viruses

④Don't know

8. What is the correct way to read body temperature with a glass thermometer? **□**

①Read the mercury end of the handheld thermometer horizontally

②Read the glass end of the handheld thermometer vertically

③Read the glass end of the handheld thermometer horizontally

④Don't know

9. Being bitten by a dog on the skin, but not serious, which is the right thing to do? **□**

①Conduct self-treatment and dressing

②Clean the wound and get vaccinated for rabies as soon as possible

③Leave the wound untreated if it's not too severe

④Don't know

10. What should parents do when their children have symptoms such as fever and rash? **□**

Agree

Disagree

Don't know

①Seek medical attention promptly

②Suspend going to kindergarten

③Timely notify the teacher at the child's kindergarten

④Allow the child to attend kindergarten as usual

⑤Don't know

11. When sick and dead livestock are found, which of the following practices is correct? **□**

①Do not kill or process animals

②Do not sell or transport animals

③Do not consume animals

④Boiled and cooked meat can be consumed

⑤Don't know

12. When coughing or sneezing, which of the following is correct? **□**

①Cover your mouth and nose with your hand directly

②Cover your mouth and nose with a handkerchief or tissue

③Cover your mouth and nose with your elbow bent at a right angle

④Do not cover your mouth and nose

⑤Don't know

**-------------** This is the end of the investigation, thank you for your support!**--------------**

**Investigator's signature** **Supervisor's signature**

**Method of investigation**: ① self-filling ② investigator inquiry

**Survey time:** year month day

**Survey site**:
